# Correlates of Loneliness and Social Isolation among Older Adults during the COVID-19 Outbreak: A Comprehensive Assessment from a National United States Sample

**DOI:** 10.3390/geriatrics9040096

**Published:** 2024-07-19

**Authors:** Miguel G. Pica, Jason R. Grullon, Roger Wong

**Affiliations:** 1 Department of Public Health and Preventive Medicine, Norton College of Medicine, SUNY Upstate Medical University, Syracuse, NY 13210, USA; 2 Norton College of Medicine, SUNY Upstate Medical University, Syracuse, NY 13210, USA; 3 Department of Geriatrics, SUNY Upstate Medical University, Syracuse, NY 13210, USA

**Keywords:** aging, coronavirus, COVID-19, disconnection, intervention, loneliness, older adult, outbreak, pandemic, social isolation

## Abstract

This study examined the correlates of loneliness and social isolation among older adults in the United States (U.S.) during the COVID-19 outbreak. We analyzed data from the 2020 National Health and Aging Trends Study, a nationally representative sample of 3257 U.S. older adults aged 65 years and older. We analyzed and identified the sociodemographic, health, social support, and community correlates of loneliness, higher loneliness during versus before the COVID-19 outbreak, and social isolation using weighted multiple logistic regression models. About 35.2% of U.S. older adults reported loneliness during the COVID-19 outbreak, 21.9% reported higher loneliness compared to before the COVID-19 outbreak, and 32.8% were socially isolated during the outbreak. Correlates for increased odds of loneliness included female gender, higher education, physical activity, depression, anxiety, functional limitations, and virtual communication access (only for higher loneliness during COVID-19 outbreak). Correlates for increased odds of social isolation included higher age, non-Hispanic Black, Hispanic, higher number of household children, and metropolitan residence. Our findings provide insights into evidence-based approaches to address social disconnection among U.S. older adults. The wide range of sociodemographic, health, social support, and community correlates identified in this study warrants multifaceted interventions that traverse individual, community, and societal levels to address the loneliness and social isolation epidemic.

## 1. Introduction

In January 2020, the COVID-19 outbreak was declared a public health emergency by the United States (U.S.) [1]. Shortly after, localized outbreaks resulted in a global pandemic. As a result, governments around the world implemented infection control measures [2], such as social distancing [3] and closing close-contact spaces [4]. Unfortunately, these measures led to social disconnection for many, detrimentally impacting their mental health [5]. In May 2023, the U.S. Surgeon General remarked that the rates of loneliness and social isolation have reached extremes and declared it a national epidemic [6].

Multiple studies have emphasized the need for a clear distinction between loneliness and social isolation due to the lack of a strong correlation between both constructs [7,8,9,10,11]. As defined by the National Academies of Sciences, Engineering, and Medicine (NASEM), loneliness is the distressing subjective experience that arises from perceived isolation or lack of meaningful connections [12]. Unlike loneliness, social isolation is a more objective measure of a lack of or restricted interpersonal relationships, group memberships, social roles, and interaction [13]. While the detrimental effects of social isolation are documented across all age groups, the COVID-19 pandemic positioned U.S. older adults in a precarious situation, compounding multi-dimensional stressors on mental, emotional, and physical well-being [14]. Given that older adults were among the first groups to receive stringent social distancing directives, they experienced a more prolonged social isolation, extending beyond their quarantine period compared to the general population [15].

Researchers and multiple scientific organizations use the term “social connection”, encompassing both loneliness and social isolation [12]. While some research has explored the effects of social connection on older adults during the COVID-19 pandemic, a May 2023 advisory from the U.S. Surgeon General noted the paucity of studies that have examined multiple co-existing components of social connection, including loneliness and social isolation, within the same sample [13]. To our knowledge, this is the first study to use a theory-based approach with a nationally representative sample of U.S. older adults to comprehensively explore a range of sociodemographic, health, social support, and community factors associated with (1) loneliness during the COVID-19 outbreak, (2) increased levels of loneliness during the pandemic compared to pre-pandemic, and (3) social isolation throughout the pandemic. In this study’s context, we have used “loneliness” and “social isolation” when data are specific to these terms, while using “social connection” when referring to both constructs collectively.

## 2. Materials and Methods

### 2.1. Data Source

We utilized data from the National Health and Aging Trends Study (NHATS), a nationally representative sample of U.S. Medicare beneficiaries aged 65 years and older. Since 2011, the NHATS has collected annual data prospectively. In 2020, the NHATS cohort completed a COVID-19 supplemental questionnaire, and most responses were completed in July or August 2020 [16]. There were 3257 respondents, representing our unweighted sample size.

### 2.2. Loneliness Variables

The first loneliness variable is from the question, “During the COVID-19 outbreak, in a typical week, how often have you felt lonely?”. Responses were re-coded into a binary variable: yes (every, most, or some days) or no (rarely or never).

The second loneliness variable is a relative measure comparing loneliness before and during the COVID-19 outbreak, which we summarized as “higher pandemic loneliness”. Specifically, the participant was asked a follow-up to the first loneliness question, “Is this more often, less often, or about the same as a typical week before the COVID-19 outbreak started?”. Responses were re-coded into a binary variable: yes (more often) or no (less often, or about the same).

### 2.3. Social Isolation Variable

Previous research has utilized the Berkman–Syme Social Network Index to develop an NHATS-equivalent social isolation variable with good convergent and divergent validity [17]. This social isolation measure consists of six items, with the absence of four or more indicating social isolation: (1) marriage or partner, (2) family to talk about important things with, (3) friend to talk about important things with, (4) visiting friends or family, (5) attending religious service, and (6) participating in clubs or organized activities.

### 2.4. Sociodemographic, Health, Social Support, and Community Variables

We utilized the NASEM theoretical framework to select sociodemographic, health status, social support, and community correlates of loneliness and social isolation [12].

Sociodemographic correlates included age, race and ethnicity (non-Hispanic White [hereafter, White], non-Hispanic Black [hereafter, Black], Hispanic, non-Hispanic Asian [hereafter, Asian], or Other), gender (male or female), total income, educational level, and in-person paid employment during the COVID-19 outbreak.

Health correlates included body mass index (BMI), physical activity (walking for exercise or vigorous physical activity), and history of either diabetes, heart disease, stroke, cancer, or hypertension aggregated into a composite number of chronic diseases (0, 1, 2, or 3+), hearing impairment (deaf/use hearing device), vision impairment (blind/wear corrective devices), major depressive disorder, generalized anxiety disorder, cigarette smoker, alcohol drinker, activities of daily living (ADL) limitations (none or at least one: bathing, dressing, toileting, transferring, or feeding), and sleep quality (poor, fair, or good).

Major depressive disorder was categorized using a cutoff of 3+ on the PHQ-2 for the frequency (1 = not at all, 2 = several days, 3 = more than half of days, 4 = nearly every day) in the last month of two items: (1) had little interest or pleasure in doing things, and (2) felt down, depressed, or hopeless. Generalized anxiety disorder was categorized using a cutoff of 3+ on the GAD-2 for the frequency (1 = not at all, 2 = several days, 3 = more than half of days, 4 = nearly every day) in the last month of two items: (1) felt nervous, anxious, or on edge, and (2) been unable to stop or control worrying.

Social support correlates included marital status, number of children in the household (0, 1, 2+), virtual communication access (phone calls, email/texts/social media) and video calls (e.g., Zoom and FaceTime), physical communication access (contact with family and friends in-person), attendance of religious services, and if they either volunteered or attended club activities in-person.

Community correlates included transportation access (access to driving a car, ride from family/friend/paid help, van/shuttle, public transit, or taxi), metropolitan residence (metro or non-metro), and residential setting (community-dwelling or residential care/nursing home).

### 2.5. Analysis Plan

Given that social isolation was binary, the two loneliness measures were also dichotomized to facilitate comparisons across the three outcomes. Therefore, three multiple logistic regression models were utilized to examine correlates for loneliness, higher pandemic loneliness, and social isolation. Marital status, religious service, and volunteer/club activity variables were removed in the social isolation regression model, as each is a component of the social isolation measure [17]. The average variance inflation factor ranged from 1.59 to 1.62 across all regression models, indicating no risk for multicollinearity. Survey sampling weights were applied in all analyses to ensure that findings were generalizable to U.S. older adults nationally. To maximize the full number of respondents in the data set and minimize bias because of missing data (23–24% across all models), multiple imputation by chained equations (MICE) generated 100 imputed data files with 10 iterations each. All analyses were performed in Stata 18 with two-tailed tests at a 0.05 significance level.

## 3. Results

### 3.1. Sample Characteristics

After applying sampling weights, the 3257 NHATS respondents represented 26,229,876 U.S. older adults. Complete sample characteristics are shown in Table 1. The mean age was 78.4 ± 6.3 years old, with a slight majority being female (55.5%), and most self-identified as White (83.0%). During the COVID-19 outbreak, only 7.3% of older adults were employed. Most of the participants had at least one chronic disease (85.6%), with a little more than half having at least two or more chronic diseases (51.7%). About 11.1% met the criteria for depression, and 8.5% for anxiety. While 77.4% of the respondents had virtual communication access and 38.3% had physical communication access, about 35.2% of the respondents felt lonely during the COVID-19 outbreak, 21.9% felt more lonely during the COVID-19 outbreak compared to before, and 32.8% met the criteria for social isolation during the COVID-19 outbreak.

### 3.2. Loneliness Correlates

The left column in Table 2 presents the sociodemographic, health, social support, and community correlates of loneliness using a weighted multiple logistic regression. For sociodemographic correlates, Black older adults had 35% lower adjusted odds of experiencing loneliness during the COVID-19 outbreak than White older adults [aOR = 0.65 (95% CI: 0.46–0.92), *p* = 0.016]. Females had 86% higher odds of loneliness than males [aOR = 1.86 (95% CI: 1.44–2.39), *p* < 0.001]. Older adults with a college degree had 82% higher odds of loneliness than those with less than a high school education [aOR = 1.82 (95% CI: 1.22–2.70), *p* = 0.004].

For health correlates, older adults who were physically active had 53% higher odds of loneliness [aOR = 1.53 (95% CI: 1.24–1.89, *p* < 0.001]. Older adults with depression had 187% higher odds of loneliness [aOR = 2.87 (95% CI: 2.07–3.98), *p* < 0.001]. Similarly, older adults with anxiety had 173% significantly higher odds of loneliness [aOR = 2.73 (95% CI: 1.82–4.12), *p* < 0.001]. There was an inverse association between quality of sleep and loneliness; good sleep quality was associated with 82% lower odds of loneliness compared to poor sleep quality [aOR = 0.18 (95% CI: 0.11–0.29), *p* < 0.001]. ADL limitations were associated with 66% higher odds of loneliness [aOR = 1.66 (95% CI: 1.18–2.34), *p* = 0.004].

For social support correlates, we found that married older adults had a significant 49% lower odds of loneliness [aOR = 0.51 (95% CI: 0.41–0.64), *p* < 0.001]. In addition, respondents with only one child had 30% lower odds of loneliness compared to those who did not have children [aOR = 0.70 (95% CI: 0.52–0.95) *p* = 0.023]. No community factors were significantly associated with experiencing loneliness during the COVID-19 outbreak.

### 3.3. Higher Pandemic Loneliness Correlates

The middle column in Table 2 presents the sociodemographic, health, social support, and community correlates for higher pandemic loneliness during the COVID-19 outbreak. For sociodemographic correlates, Black older adults had 35% significantly lower odds of experiencing higher loneliness during the COVID-19 outbreak [aOR = 0.65 (95% CI: 0.43–0.98), *p* = 0.041]. Females had 103% higher odds of experiencing pandemic loneliness [aOR = 2.03 (95% CI: 1.55–2.66), *p* < 0.001]. Older adults with a college degree had higher odds for higher loneliness by a significant 148% compared to those with less than a high school education [aOR = 2.48 (95% CI: 1.44–4.29), *p* = 0.002]. This was also the same for older adults with a high school degree, with significantly higher odds of 72% for higher pandemic loneliness [aOR = 1.72 (95% CI: 1.04–2.85), *p* = 0.037]. Employed older adults had 52% lower odds for higher pandemic loneliness [aOR = 0.48 (95% CI: 0.23–0.98), *p* = 0.044].

For health correlates, older adults who were physically active had 38% higher odds for higher pandemic loneliness [aOR = 1.38 (95% CI: 1.08–1.76), *p* = 0.010]. Older adults with anxiety had 92% higher odds for higher pandemic loneliness [aOR = 1.92 (95% CI: 1.27–2.89), *p* = 0.003]. Older adults with good sleep quality had 68% lower odds for higher pandemic loneliness than those with poor sleep quality [aOR = 0.32 (95% CI: 0.20–0.51), *p* < 0.001]. This pattern continued for older adults with fair sleep quality, with 44% lower odds for higher pandemic loneliness [aOR = 0.56 (95% CI: 0.35–0.90), *p* = 0.017].

For social support correlates, we found that married older adults had 32% significantly lower odds for higher pandemic loneliness [aOR = 0.68 (95% CI: 0.51–0.90), *p* = 0.007]. Older adults with only one child had 37% lower odds for higher pandemic loneliness than those without children [aOR = 0.63 (95% CI: 0.46–0.88), *p* = 0.007]. Older adults with access to virtual communication resources had 64% increased odds of higher pandemic loneliness [aOR = 1.64 (95% CI: 1.19–2.27), *p* = 0.003]. No community correlates were significantly associated with experiencing higher pandemic loneliness.

### 3.4. Social Isolation Correlates

The right column in Table 2 presents the sociodemographic, health, social support, and community correlates for social isolation during the COVID-19 outbreak. For sociodemographic correlates, for every one-year increase in age, the odds of social isolation increased significantly by 3% [aOR = 1.03 (95% CI: 1.02–1.05), *p* < 0.001]. Black older adults had 45% higher odds for social isolation [aOR = 1.45 (95% CI: 1.07–1.97), *p* = 0.017]. This pattern was similar for Hispanic older adults, with 76% higher odds of social isolation [aOR = 1.76 (95% CI: 1.07–2.91), *p* = 0.028]. There was also an inverse association between income and social isolation [aOR = 0.52 (95% CI: 0.44–0.60), *p* < 0.001]. For health correlates, physically active older adults had 37% significantly lower odds for social isolation [aOR = 0.63 (95% CI: 0.49–0.81), *p* < 0.001]. 

For social support correlates, older adults with only one child had 47% higher odds of social isolation than those without children. These higher odds for social isolation continued as the number of children in the household increased. Older adults with two or more children had significantly increased odds of 148% for social isolation compared to those without children [aOR = 2.48 (95% CI: 1.05–5.84), *p* = 0.002]. Older adults with access to virtual communication resources had 34% lower odds of social isolation [aOR = 0.66 (95% CI: 0.51–0.85), *p* = 0.002].

For community correlates, older adults residing in a metropolitan residence had 42% increased odds for social isolation [aOR = 1.42 (95% CI: 1.11–1.82), *p* = 0.007]. Conversely, older adults with transportation access had 65% significantly lower odds for social isolation [aOR = 0.35 (95% CI: 0.15–0.83), *p* = 0.019].

## 4. Discussion

Our study indicated that numerous sociodemographic, health, social support, and community correlates were associated with social connection during the COVID-19 outbreak among U.S. older adults. Loneliness and higher pandemic loneliness were associated with several sociodemographic, health, and social factors, while social isolation was additionally associated with several community correlates. Unsurprisingly, many correlates significantly associated with loneliness were not associated with social isolation, and vice versa. However, with correlates significant for loneliness and social isolation, there were opposing directional relationships.

Most of our findings on loneliness are consistent with prior literature, which has noted females [3,7,9,18,19], mental health disorders such as depression and anxiety [7,9,20,21,22,23], and functional limitations [9] as potential risk factors for loneliness. However, we also found several protective factors, such as good sleep quality, being married, and having children in the household [3,19].

Previous studies have found that Black older adults experience loneliness more frequently [3,24,25]. In contrast, we found that Black older adults had lower odds of experiencing loneliness during the COVID-19 outbreak. A possible explanation is that our model did not include any neighborhood measures, such as social cohesion, which prior research has shown is significantly lower among Black older adults [26].

Similarly, higher education was associated with increased odds of loneliness. A potential reason for this may be due to the shift in their social networks during the pandemic. Higher education is often associated with increased occupational and social mobility, and many high-income earners suddenly had to shift from daily in-person interactions to remote work. The Pew Research Center noted that 65% of adults employed in telework had a bachelor’s degree or higher, and workers who pivoted from in-person to telework were found to be 60% less connected to their coworkers [27].

We also observed an unexpected positive association between physical activity and loneliness. Older adults often rely on established routines for their social interactions and physical activities [28]. However, the implementation of lockdown mandates, social distancing, and business closures disrupted these routines, ultimately creating difficulties for older adults in maintaining their usual physical activity and social engagement [28], which may have led to perceptions of disconnection and increased loneliness.

Regarding social isolation, higher age, identifying as Black or Hispanic, lower income, physical inactivity, children in the household, no virtual communication access, metropolitan area residence, and no transportation access were independent risk factors among U.S. older adults. These findings were largely consistent with a systematic review using objective measures for social isolation among older adults [29]. However, our findings should be considered in the context of the pandemic situation.

For instance, Black and Hispanic community-dwelling older adults exhibited lower rates of social isolation in the pre-pandemic era than their White counterparts [30], while the reverse was observed during the pandemic, likely due to changes in social network size, composition, and density as a major contributor to social isolation [31]. Moreover, restrictions on in-person religious gatherings likely constrained the non-kin ties that Black and Hispanic older adults tend to establish more often through religious congregations leading to an increased sense of social disconnection [32,33]. Given prior research that has noted elevated odds of COVID-19 diagnosis for Black and Hispanic older adults [34,35], it would also be worthwhile to explore whether mandated quarantines may have contributed to higher odds of social isolation.

The shift toward video conferencing may have isolated low-income older adults with insufficient resources and/or familiarity to access high-speed internet and digital technology [36]. Low-income older adults also tend to report less available social support than their higher-income counterparts [37]. Meanwhile, pandemic-related financial hardship could apply greater financial strain and psychosocial stress [38]. These underscore the disproportionately high impact of the COVID-19 pandemic on marginalized communities, such as racial and ethnic minorities and low-income households. In addition to the higher odds of social isolation during the pandemic, minority and low-income older adults experienced higher odds of COVID-19 diagnosis despite their higher adherence to mitigation behaviors [39].

Research into the protective factors against social isolation among U.S. older adults during the pandemic is limited. However, consistent with findings of international studies, our analysis identified regular physical activity, access to virtual communication, higher income, and available transportation as protective factors. As discussed in the context of loneliness, older adults tend to blend regular physical exercise with established routines, including maintaining social connections, which may contribute to a lower risk of social isolation [40]. Similarly, access to virtual communication platforms allows older adults to connect with friends, family, and acquaintances, mitigating the likelihood of social isolation during the pandemic [41]. Additionally, higher income and access to transportation among older adults allows for greater social engagement in clubs, organizations, or leisure activities, which can foster a sense of belonging or social connectedness [42].

There were several limitations to our study. First, we analyzed data within a short window, inhibiting causal inferences. Second, even though validated loneliness scales are available in other research, the NHATS only collects data on self-reported loneliness due to the long questionnaire length. Third, mortality was relatively high among older populations during the initial stages of the pandemic, which may contribute to bias in who could participate in the study. Despite these limitations, our study is an important contribution to the literature as it analyzes a large variety of theory-driven correlates for loneliness and social isolation using a nationally representative U.S. sample. Finally, due to the rarity of a global pandemic and the emergence of innovative technologies, our study also addresses limitations in previous research by investigating whether virtual versus physical-mediated communication was associated with social connection [13].

## 5. Conclusions

Our investigation into the intricate correlates of loneliness and social isolation provides insights into possible evidence-based approaches to address the growing social disconnection crisis among older adults. The wide range of sociodemographic, health, social support, and community correlates identified in this study warrants multifaceted interventions that traverse individual, community, and societal levels. These multi-level efforts would include individual behaviors from socially disconnected older adults, family and friends, health providers, voluntary organizations, government, health authorities, and charities [12]. We also found some correlates for loneliness and social isolation to be mutually exclusive and others with opposing directional relationships. Therefore, future research must examine both constructs separately and establish standard international measurements and clear definitions for both social isolation and loneliness that are cross-culturally valid [11]. Our findings can guide strategies and policies beyond the current COVID-19 pandemic.

## Figures and Tables

**Table 1:** Weighted sample characteristics for participants in the National Health and Aging Trends Study.

Variables		Mean (SD) or % (n) ^a^
Sociodemographic	Age (range 70–112)	78.4 (6.3)
	Race and Ethnicity	
	White	83.0% (21.8)
	Black	6.5% (1.7)
	Hispanic	5.1% (1.3)
	Asian	2.0% (0.5)
	Other	3.5% (0.9)
	Female	55.5% (14.5)
	Income (thousands of U.S. dollars)	67.9 (68.6)
	Highest Level of Education	
	<High School	11.4% (2.9)
	High School	47.8% (12.3)
	College	40.8% (10.5)
	Employment Status	
	Not employed	92.7% (24.3)
	Employed	7.3% (1.9)
Health	Body Mass Index	27.9 (5.6)
	Physically Active	74.6% (19.6)
	Hearing Impairment	20.4% (5.3)
	Vision Impairment	62.1% (16.3)
	Major Depressive Disorder	11.1% (2.9)
	Generalized Anxiety Disorder	8.5% (2.2)
	Cigarette Smoker	4.2% (1.1)
	Alcohol Drinker	41.6% (10.2)
	Sleep Quality	
	Poor	7.1% (1.8)
	Fair	35.1% (9.0)
	Good	57.8% (14.8)
	Number of Chronic Diseases ^b^	
	0	14.5% (3.7)
	1	33.9% (8.8)
	2	33.1% (8.6)
	3+	18.6% (4.8)
	Activities of Daily Living Limitations	13.0% (3.4)
Social Support	Marital Status (Married)	55.5% (14.6)
	Number of Children in Household	
	0	83.7% (22.0)
	1	14.4% (3.8)
	2+	1.8% (0.5)
	Virtual Communication Access ^c^	77.4% (20.3)
	Physical Communication Access	38.3% (10.0)
	Attend Religious Services	11.3% (3.0)
	Attend Volunteer/Club Activities	11.7% (3.1)
Community	Residential Care/Nursing Home	5.7% (1.5)
	Metropolitan Residence	81.6% (21.4)
	Transportation Access	96.9% (25.4)
Social Connection	Loneliness	35.2% (8.7)
	Higher Pandemic Loneliness	21.9% (5.3)
	Social Isolation	32.8% (8.0)

^a^ All frequencies are in millions. ^b^ Chronic diseases are self-reported by respondents with “yes” to ever being diagnosed with diabetes, heart disease, cancer, stroke, or hypertension. ^c^ Communication methods included phone calls, email/texts/social media (Facebook), and video calls (Zoom, FaceTime, or another online platform).

**Table 2:** Weighted logistic regression examining correlates for loneliness, higher pandemic loneliness, and social isolation during the COVID-19 outbreak.

Variables	Loneliness		Higher Pandemic Loneliness		Social Isolation	
	aOR (95% CI)	*p*-Value	aOR (95% CI)	*p*-Value	aOR (95% CI)	*p*-Value
**Sociodemographic**						
Age	1.00 (0.99–1.02)	0.662	0.98 (0.96–1.00)	0.071	1.03 (1.02–1.05)	**<0.001**
Race and Ethnicity						
White	(Ref)	(Ref)	(Ref)	(Ref)	(Ref)	(Ref)
Black	0.65 (0.46–0.92)	**0.016**	0.65 (0.43–0.98)	**0.041**	1.45 (1.07–1.97)	**0.017**
Hispanic	0.91 (0.53–1.57)	0.739	0.79 (0.44–1.41)	0.417	1.76 (1.07–2.91)	**0.028**
Asian	1.30 (0.54–3.14)	0.555	0.94 (0.35–2.49)	0.893	1.99 (0.71–5.60)	0.186
Other	0.63 (0.33–1.23)	0.171	0.55 (0.17–1.76)	0.304	1.13 (0.59–2.19)	0.702
Female	1.86 (1.44–2.39)	**<0.001**	2.03 (1.55–2.66)	**<0.001**	1.11 (0.89–1.39)	0.348
Income (log U.S. dollars)	0.94 (0.81–1.10)	0.441	1.16 (0.96–1.40)	0.126	0.52 (0.44–0.60)	**<0.001**
Highest Level of Education						
<High School	(Ref)	(Ref)	(Ref)	(Ref)	(Ref)	(Ref)
High School	1.39 (0.97–1.98)	0.069	1.72 (1.04–2.85)	**0.037**	0.82 (0.59–1.12)	0.205
College	1.82 (1.22–2.70)	**0.004**	2.48 (1.44–4.29)	**0.002**	0.84 (0.54–1.30)	0.426
Employed	0.66 (0.42–1.03)	0.067	0.48 (0.23–0.98)	**0.044**	1.07 (0.67–1.70)	0.783
**Health**						
Body Mass Index	1.00 (0.99–1.02)	0.729	1.00 (0.98–1.02)	0.901	0.98 (0.96–1.00)	0.073
Physically Active	1.53 (1.24–1.89)	**<0.001**	1.38 (1.08–1.76)	**0.010**	0.63 (0.49–0.81)	**<0.001**
Hearing Impairment	0.93 (0.73–1.18)	0.548	0.99 (0.75–1.31)	0.948	0.95 (0.75–1.22)	0.697
Vision Impairment	0.95 (0.76–1.19)	0.676	0.94 (0.75–1.20)	0.628	0.96 (0.77–1.19)	0.717
Major Depressive Disorder	2.87 (2.07–3.98)	**<0.001**	1.17 (0.85–1.61)	0.334	1.29 (0.93–1.78)	0.120
Generalized Anxiety Disorder	2.73 (1.82–4.12)	**<0.001**	1.92 (1.27–2.89)	**0.003**	1.02 (0.70–1.50)	0.909
Cigarette Smoker	1.26 (0.77–2.05)	0.343	0.92 (0.53–1.59)	0.749	1.14 (0.74–1.76)	0.551
Alcohol Drinker	1.08 (0.84–1.38)	0.544	1.30 (0.92–1.84)	0.128	1.05 (0.82–1.34)	0.690
Sleep Quality						
Poor	(Ref)	(Ref)	(Ref)	(Ref)	(Ref)	(Ref)
Fair	0.46 (0.28–0.75)	**0.002**	0.56 (0.35–0.90)	**0.017**	0.95 (0.60–1.49)	0.808
Good	0.18 (0.11–0.29)	**<0.001**	0.32 (0.20–0.51)	**<0.001**	0.85 (0.53–1.37)	0.501
Number of Chronic Diseases ^a^						
0	(Ref)	(Ref)	(Ref)	(Ref)	(Ref)	(Ref)
1	1.16 (0.83–1.62)	0.381	1.09 (0.73–1.61)	0.674	0.85 (0.59–1.23)	0.389
2	1.14 (0.78–1.66)	0.501	1.19 (0.80–1.79)	0.381	1.05 (0.73–1.51)	0.790
3+	1.28 (0.89–1.85)	0.183	1.38 (0.92–2.07)	0.118	1.08 (0.74–1.58)	0.692
Activities of Daily Living Limitations	1.66 (1.18–2.34)	**0.004**	1.12 (0.77–1.63)	0.557	0.84 (0.60–1.16)	0.281
**Social Support**						
Married	0.51 (0.41–0.64)	**<0.001**	0.68 (0.51–0.90)	**0.007**	-	-
Number of Children in Household						
0	(Ref)	(Ref)	(Ref)	(Ref)	(Ref)	(Ref)
1	0.70 (0.52–0.95)	**0.023**	0.63 (0.46–0.88)	**0.007**	1.47 (1.08–2.00)	**0.014**
2+	0.53 (0.26–1.06)	0.073	0.5 (0.18–1.43)	0.192	2.48 (1.05–5.84)	**0.039**
Virtual Communication Access ^b^	1.06 (0.84–1.34)	0.610	1.64 (1.19–2.27)	**0.003**	0.66 (0.51–0.85)	**0.002**
Physical Communication Access	0.95 (0.78–1.16)	0.605	1.16 (0.90–1.49)	0.238	0.85 (0.67–1.08)	0.179
Attend Religious Services	0.78 (0.57–1.07)	0.123	0.84 (0.60–1.19)	0.330	-	-
Attend Volunteer/Club Activities	0.78 (0.57–1.07)	0.118	0.94 (0.68–1.29)	0.701	-	-
**Community**						
Residential Care/Nursing home	1.23 (0.83–1.81)	0.299	1.30 (0.81–2.08)	0.265	1.46 (0.96–2.24)	0.079
Metropolitan Residence	0.92 (0.70–1.21)	0.539	1.21 (0.85–1.71)	0.283	1.42 (1.11–1.82)	**0.007**
Transportation Access	0.66 (0.31–1.41)	0.272	1.36 (0.57–3.28)	0.473	0.35 (0.15–0.83)	**0.019**
**Model Significance**	F(34,52) = 18.54, *p* < 0.001		F(34,52) = 8.83, *p* < 0.001		F(31,52) = 20.25, *p* < 0.001	

Note: aOR = adjusted odds ratio. Bold indicates statistically significant (*p* < 0.05). ^a^ Chronic diseases are self-reported by respondents with “yes” to ever being diagnosed with diabetes, heart disease, cancer, stroke, or hypertension. ^b^ Communication methods included phone calls, email/texts/social media (Facebook), and video calls (Zoom, FaceTime, or another online platform).

## Data Availability

This study uses non-public sensitive data, which may be obtained through an application from the National Health and Aging Trends Study (https://nhats.org/) accessed on 17 April 2023.
